# The stability of paintings and the molecular structure of the oil paint polymeric network

**DOI:** 10.1038/s41598-021-93268-8

**Published:** 2021-07-09

**Authors:** Francesca Nardelli, Francesca Martini, Judith Lee, Anna Lluvears-Tenorio, Jacopo La Nasa, Celia Duce, Bronwyn Ormsby, Marco Geppi, Ilaria Bonaduce

**Affiliations:** 1 Department of Chemistry and Industrial Chemistry, University of Pisa, Via Giuseppe Moruzzi 13, 56124 Pisa, Italy; 2 Centro Per L’Integrazione Della Strumentazione Scientifica Dell’Università Di Pisa (CISUP), Lungarno Pacinotti 43, 56126 Pisa, Italy; 3 Conservation Department, Tate, Millbank, London, SW1P 4RG UK

**Keywords:** Materials chemistry, Mass spectrometry, NMR spectroscopy

## Abstract

A molecular-level understanding of the structure of the polymeric network formed upon the curing of air-drying artists’ oil paints still represents a challenge. In this study we used a set of analytical methodologies classically employed for the characterisation of a paint film—based on infrared spectroscopy and mass spectrometry—in combination with solid state NMR (SSNMR), to characterise model paint layers which present different behaviours towards surface cleaning with water, a commonly applied procedure in art conservation. The study demonstrates, with the fundamental contribution of SSNMR, a relationship between the painting stability and the chemical structure of the polymeric network. In particular, it is demonstrated for the first time that a low degree of cross-linking in combination with a high degree of oxidation of the polymeric network render the oil paint layer sensitive to water.

## Introduction

The curing of a drying oil—a glycerolipid based on polyunsaturated fatty acids—involves autoxidative radical chain reactions, which are light initiated, and metal catalysed^1–6^. Upon curing, fatty acids in triglycerides undergo several transformations, which entail the formation of epoxy, hydroxyl, oxo and carbonyl moieties, as well as new C–C and C–O–C bonds^1,4,6,7^. Small oxidised molecules are formed by oxidative degradation of the fatty acid chains upon evolution of peroxyl and alkoxyl moieties^4,6^, and are quickly lost by evaporation^8^. With time, hydrolysis of ester bond takes place^9–11^ and, depending on the nature of the pigment and additives, formation of metal soaps occurs^12^. As a result, the organic molecular composition of an oil paint layer evolves from polyunsaturated triglycerides to a significantly more complex system, whose composition evolves over years, even centuries, entailing the simultaneous presence of free fatty acids, free dicarboxylic acids, mono-, di- and triglycerides, and cross-linked fractions^2,3,5,7,10,11,13–17^. In addition, when metal soaps are formed, carboxyl moieties are bound to metal cations, in variable proportions depending on the age of the paint, the environmental conditions^18,19^, and the nature of the pigment^20^, leading to the formation of free metal soaps and ionomer-like networks^21–23^.

The detection of a drying oil in a painting is, in general, relatively straightforward, and can be achieved using a variety of analytical approaches, including, most commonly, those based on FTIR^24–26^, GC-MS^27^, Py-GC-MS^28^, HPLC-MS^29^.

On the other hand, the chemical speciation of the different fractions in a painting sample requires different analytical approaches. Free acidic moieties are detected by IR spectroscopy^30^. Free fatty and dicarboxylic acids can be determined qualitatively and quantitatively by GC-MS based techniques^11,13,31,32^, HPLC-MS^14^ and flow injection analysis coupled with ESI-MS^33^. Metal soaps have been extensively studied with spectroscopic techniques which are helping to distinguish between free metal soaps and those belonging to the ionomeric network^12,20,21,23,30,34–40^. Free metal soaps may also be determined by using mass spectrometry^32,41^. HPLC-MS based methods are the methods of choice for glyceride profiling in paintings^29^, and flow injection analysis coupled with ESI–MS can help visualise the distribution of glycerides and more abundant oligomers in a paint layer^33,42^. While spectroscopic techniques allow the bulk of the sample or its surface to be analysed, based on the instrumental set-up used, GC-MS, HPLC-MS and ESI-MS give only information on the soluble/hydrolysable fraction of the oil matrix.

Characterisation of the cross-linked fractions is significantly more challenging, if possible at all. Fatty and dicarboxylic acids covalently bound to the polymeric network through ester bonds can be analysed by GC-MS after hydrolysis, which can be achieved both in acidic and alkaline environments^27,28^. Analytical pyrolysis is the only MS based technique which allows the analysis of the whole organic fraction of the sample^43^, including the cross-linked network, although data interpretation is not straightforward and only qualitative and semiquantitative information can be obtained^14,44^. Attenuated total reflection Fourier-transform infrared (ATR-FTIR) spectroscopy was recently used to determine the concentration of COOH groups covalently linked to an oil polymer network^13^. This study elegantly demonstrated that the oxidation of a paint film produces a high number of new acidic moieties which are covalently bound to the polymeric network—one COOH group per triacylglyceride—within linseed oil and zinc oxide based model paints^13^.

**Figure 1:**
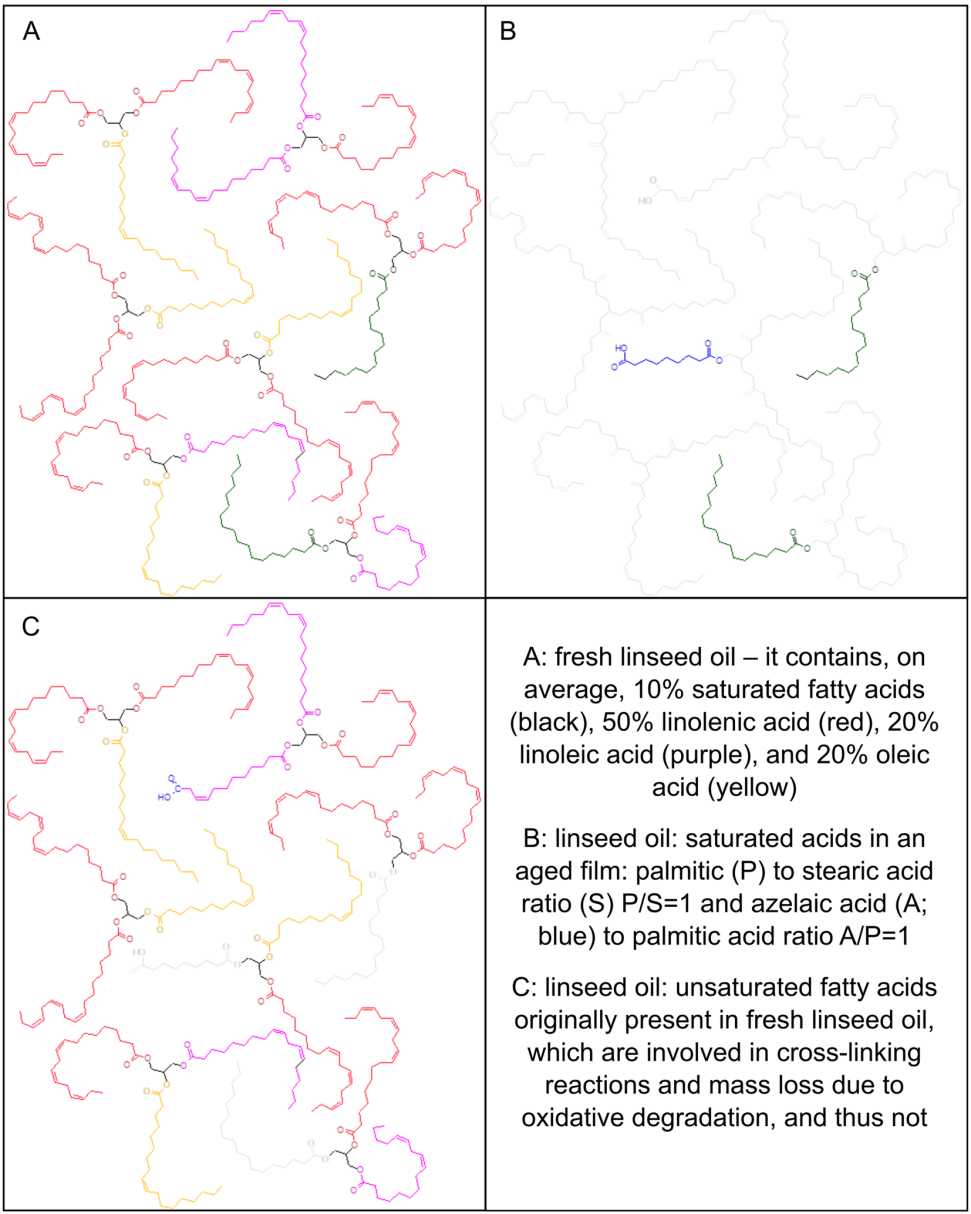
Triglyceride composition of linseed oil, and its evolution in a paint film.

Achieving a detailed molecular characterisation of the oil polymeric network, determining the degree of cross-linking and oxidation, clearly remains one of the most significant analytical challenges. This represents a major shortcoming in our understanding of the chemistry of oil paints, as, in general, the cross-linked fraction of a cured and aged paint film represents—from a quantitative point of view—the majority of the organic fraction. This can easily be seen if we look at the chemical composition of—for example—linseed oil, one of the most commonly used oil paint binders. Linseed oil triglycerides are made up of linolenic acid (52–55%), palmitic acid (about 7%) and stearic acid (3–5%), oleic acid (18–23%), linoleic acid (14–17%)^45^. In a sample taken from a mature oil painting, subject to hydrolysis followed by derivatisation and GC–MS analysis, the ratio between the relative content of palmitic acid and stearic acid is about 1 (P/S = 1), the ratio between the relative content of azelaic acid (a stable oxidation product) and palmitic acid is about 1 (A/P = 1)^27^. This means that on average, saturated mono and dicarboxylic acids (excluding those arising from additives) account for about 20–25% of the organic binder in an aged paint layer (the exact amount depends on the degree of mass loss due to oxidative degradation^44^). The remaining material comprises the (macro)molecules formed from more than 80% of the unsaturated fatty acids originally constituting the oil (Fig. 1), which are not amenable to GC-MS nor HPLC-MS analysis.

In an oil painting the chemical structure of the polymeric network, which arises from the evolution of the vast majority of the unsaturated fatty acids of a drying oil, plays a fundamental role in defining the stability of a paint film, as it has recently been hypothesised in relation to certain degradation phenomena which are typical of modern oil paintings^1,14,33,46,47^.

In this study we explored the use of Solid State Nuclear Magnetic Resonance (SSNMR) spectroscopy to gain insights into the chemical composition of the organic fractions of an oil paint film, including the cross-linked network, and to relate this knowledge to the holistic properties of the paint layer. Indeed, SSNMR is particularly useful to dissect the structural and dynamic properties of polymeric materials at the molecular scale^48–51^. NMR techniques have been used to characterise and monitor a range of Cultural Heritage materials^52–55^ and have proven extremely valuable in the study of metal soaps^35,56,57^, and have occasionally been used to characterise the organic fractions of oil paintings. As thoroughly discussed elsewhere^35,58^, mobile fractions in drying oils may be analysed by liquid-state NMR^59,60^, complementing MS based studies, and swollen-state NMR may help to assess the insoluble fraction of the oil paint layers^61^. Ultrafast spinning SSNMR spectroscopy was used in recent research on two case study paintings, highlighting the potential of this technique to access all fractions—mobile and immobile—within the paint layers^58^. This technique detected the formation of C–O–C cross-links, and determined that glycerides are the main constituents of the paint mobile fraction^58^. Compared to other analytical methods, one of the major advantages of SSNMR is that this technique facilitates the analysis of whole materials without altering the native state of the samples. Various experiments can be exploited to selectively characterize regions of the sample displaying different molecular mobility, since they can be distinguished based on specific different nuclear properties (T_1_ and T_2_ relaxation times, homonuclear ^1^H–^1^H and heteronuclear ^1^H–^13^C dipolar couplings, etc*.*), without the requirement of preventive extraction procedures or chemical treatment.

In the current study, two model paint layers were prepared, from the same commercial paint—Winsor & Newton (W&N) Artists’ Oil Colour—French Ultramarine. Ultramarine blue based oil paints may present well known conservation issues. Oil paint layers containing this pigment may appear greysh and poorly coherent^62–64^, and, in modern paintings, they may show sensitivity to the action of water^1,14,46^. After initial drying of both model paint layers, one of them was subjected to accelerated aging under high relative humidity (% RH) and light^65^. High relative humidity has been shown to favour oxidation in paint films^13,33^, and resulted in the paint layer to become sensitive to the action of water during cleaning^46,66,67^. The model paint layers were thus investigated with a variety of analytical methodologies classically employed for the characterisation of a paint film—TGA, FTIR and ATR-FTIR, GC-MS, Py-GC-MS, EGA-MS, HPLC-MS—in combination with SSNMR, for a molecular characterisation of all the fractions of the oil: the soluble glyceride fraction, the hydrolysable fraction, the free fatty acids, the metal soaps, and the cross-linked network.

## Results

### Water sensitivity tests

Paint swelling and slight pigment loss was observed after 14 deionised water swab rolls applied to the surface of the artificially aged paint film. The naturally aged paint film was unaffected by swab rolling, up to the arbitrary stopping point of 50 swab rolls. For this reason, the control sample that had experienced natural ageing only, from this point onwards will be referred to as NWS (Non-Water Sensitive), and the artificially aged sample will be referred to as WS (Water Sensitive).

### FTIR and SEM-EDX

**Figure 2:**
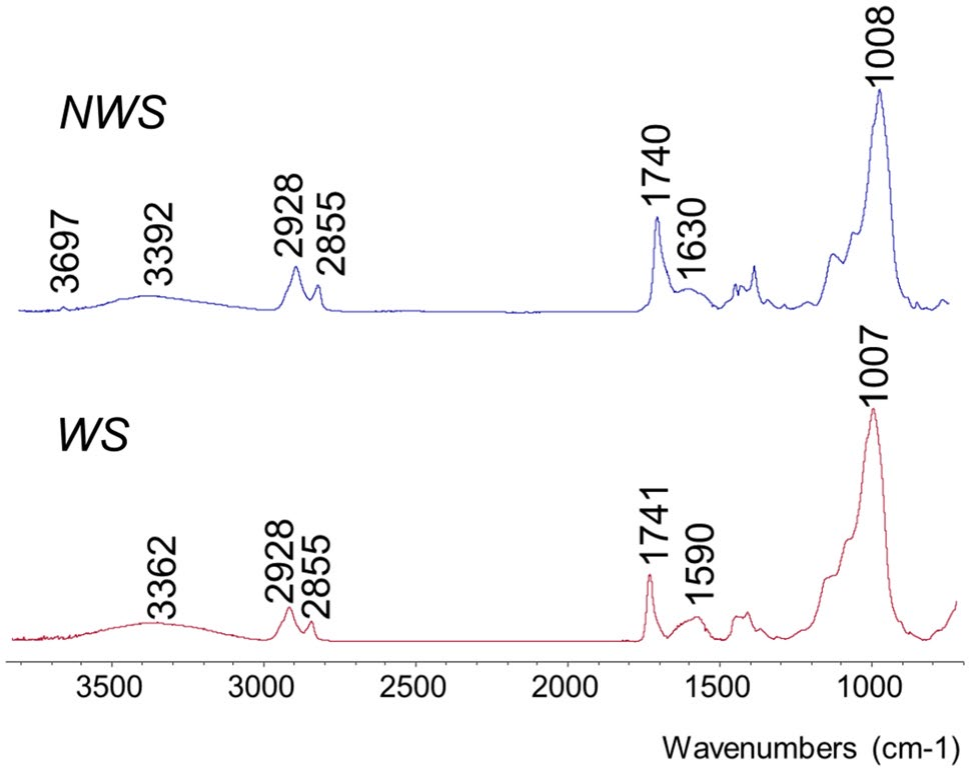
Ge-ATR spectra of control NWS sample (top) and WS sample (bottom).

Analyses were performed on KBr pellets prepared from removed sample flakes, for obtaining information on the bulk of the sample (Figure S.1), and by Ge-ATR, for characterising the sample surface (Fig. 2). Band assignment is reported in Table S.2. Elemental analysis was also performed by SEM-EDX and the EDX spectrum is reported in Figure S.2. In addition to the oil and the pigment, hydromagnesite and kaolin were identified; these are known extenders used by W&N. Kaolin is also used in the manufacture of synthetic ultramarine and it may be present by association with the pigment. The paint composition is similar to the historical 1965 formulation shown in Table S.1.

Although the 1965 formulation includes hydrated aluminium oxide, its identification using FTIR is complicated, due to the overlap of characteristic absorptions with those of the ultramarine pigment^68^. The FTIR spectra of the bulk samples were extremely similar to each other, while subtle differences were visible at the sample surface in the ATR spectra (Fig. 2). The WS surface features a slight reduction of the intensity of ester carbonyl absorption bands at 1741 and 1161 cm^−1^, accompanied by the emergence of a broad band, at 1590 cm^−1^, which may indicate the formation amorphous metal carboxylates—possibly magnesium carboxylates, originating from the reaction of acidic moieties with the hydromagnesite extender. Small amounts of metal soaps may be included in the original paint formulation, as a shoulder is visible in the FTIR spectra in the region around 1590 cm^−1^ of NWS (Figure S.1, Fig. 2), which is in agreement with the known use of magnesium stearates by W&N^69^, and its inclusion in past W&N ultramarine blue paint formulations (Table S.1). Changes to the hydromagnesite signals at 1483 and 1420 cm^−1^ ascribed to the presence of two CO_3_^2−^ environments^70^ were also observed at the paint surface, with the absorption at 1483 cm^−1^ being absent in the WS sample. This may indicate both a change in the hydration of hydromagnesite induced by the artificial ageing, and the formation of Mg metal soaps. The presence of epsomite (MgSO_4_.7H_2_O)—a water soluble salt and known cause of water sensitivity^66^, was investigated. Inorganic sulfates are characterised using IR spectroscopy by a strong S–O stretch, which is expected in the 1140–1080 cm^−1^ range^71^. Whilst a shoulder at 1096 cm^−1^ is visible in both the NWS and WS sample, a shoulder does also appear in the reference spectra for French Ultramarine pigments^72^. Hence the presence of epsomite is not evident in the infrared spectra.

### TGA

**Figure 3:**
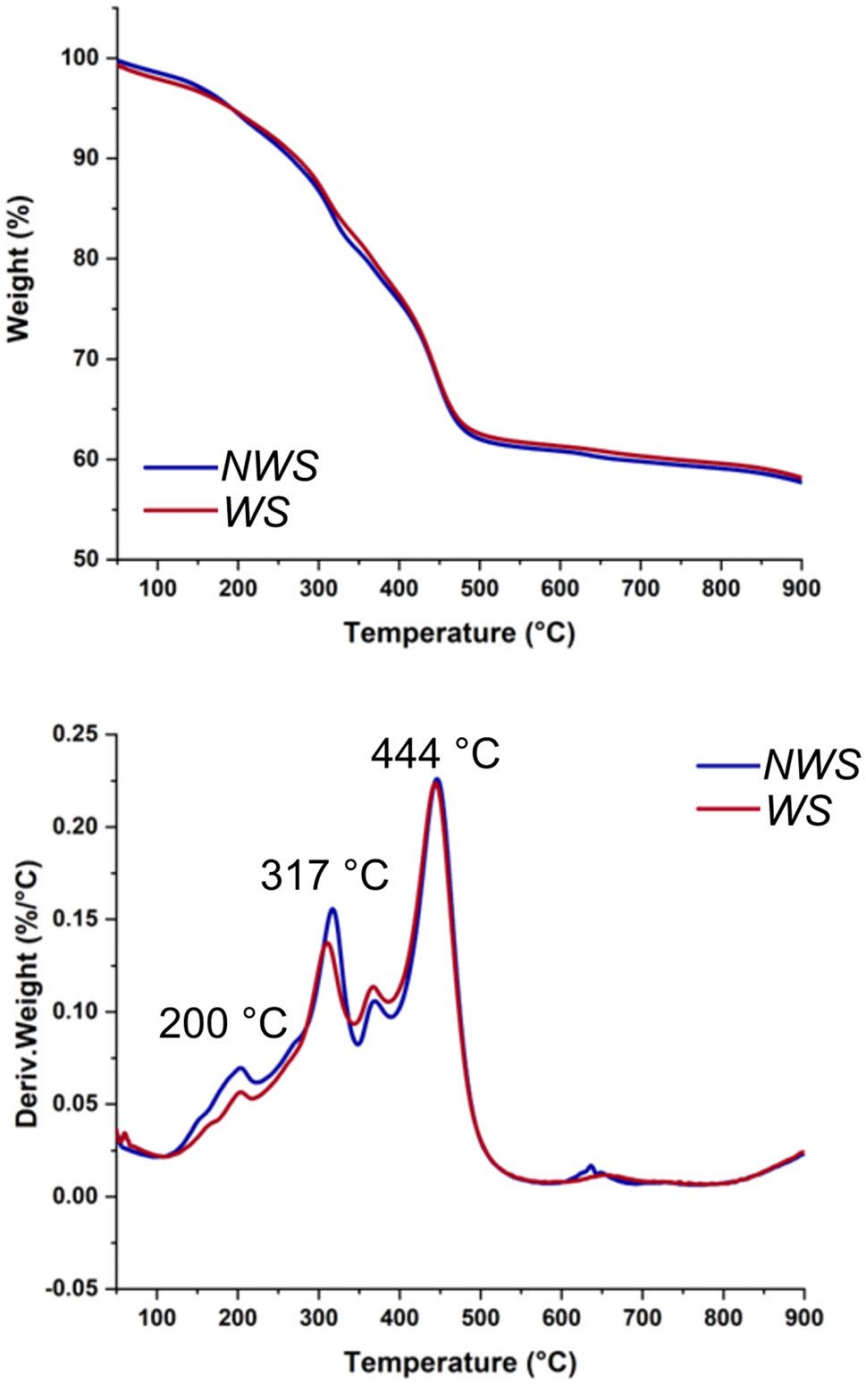
Thermogravimetric curves (top) and their derivatives (bottom) of WS and NWS samples at 10°min^−1^ under nitrogen.

Thermogravimetric curves and their derivatives are reported in Fig. 3, showing the same thermodegradative profiles for the NWS and WS samples. The mass loss at about 50 °C is due to moisture evaporation and occurs in both samples at less than 2%. In the temperature range 200–500 °C, the pyrolysis of the oil main components (mono-, di- and tri- glycerides, free fatty acids and metal soaps) occurs^65^, overlapped with that of hydromagnesite, and, possibly, hydrated aluminum oxide^73,74^ . The residue of almost 60% total weight at 550 °C is mainly due to the inorganic portion of the paints, of which the pigment is stable over the temperature range investigated (Figure S.3). These proportions concur with the relative solids to medium weight percentages stated in the 1965 paint formulation (Table S.1).

### Mass spectrometry

#### GC-MS

GC-MS was performed on the samples after saponification, hydrolysis and derivatisation^75^, in order to free fatty acids, as well as dicarboxylic acids produced upon ageing, also present as metal soaps, which are non-covalently bound to the polymeric network through C–C and C–O–C cross-links^13^. Figure S.4 reports the chromatograms of samples NWS and WS and Table S.3 data quantitation. The sample WS presents a relatively higher content of oleic acid and a relatively lower content of dicarboxylic acids, indicating that the water sensitive sample is following different curing pathways and kinetics^11,75^. The chromatograms present significant amounts of heptadecanoic acid (see Figure S.4), ascribable to paint additives (i.e. free fatty acids, metal soaps or animal fats).

#### HPLC-MS

**Figure 4:**
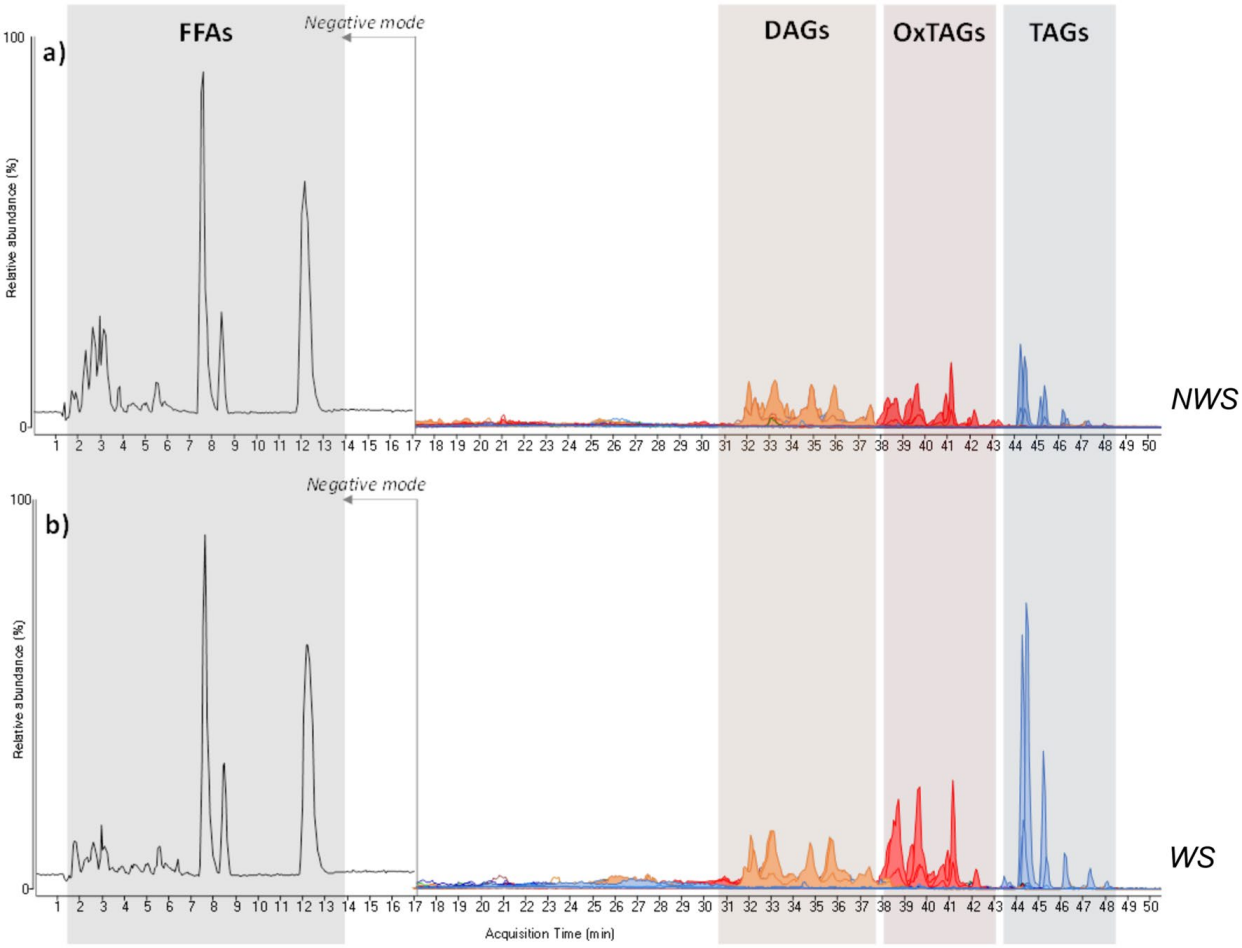
HPLC-ESI-Q-ToF extracted ion chromatograms obtained for the extracts of NWS (**a**) and WS (**b**). The two chromatograms were normalized according to the tristearin content. The complete list of all the species detected is reported in Table S.4 in the supplementary materials.

Samples were analysed by HPLC-MS after solvent extraction in order to investigate the free fatty acids and glyceride composition. Chromatograms are shown in Fig. 4.

Both the samples presented free fatty acids, and glycerides, including oxidized diglicerides (DAGs) and triglycerides (TAGs) deriving from the oxidation of acylglycerols containing linolenic and linoleic acid. The detailed list of compounds identified is reported in Table S.4. By combining the data interpretation for both the oxidized and non-oxidised species^29^ we identified a mixture of linseed oil and traces of safflower oil as the paint binder.

NWS shows higher relative amounts of free fatty acids (mainly palmitic, stearic, oleic acids), as well as oxidized species (hydroxyl derivatives of unsaturated fatty acids with 18 carbons, oxDAGs and oxTAGs) respect to the non-oxidized TAGs detected. 

Finally, heptadecanoic acid, nor free, nor bound to glycerides was detected, indicating that the heptadecanoic acid detected by GC-MS is due to added metal soaps.

### Py-GC-MS

Analytical pyrolysis with in-situ silylation was used to analyse the whole organic fraction of the sample^28,76^. The pyrolytic profiles are reported in Figure S.5. The first part of the chromatograms obtained from both samples are extremely similar and are dominated by linear short chain saturated and unsaturated fatty acids, mostly ranging between 6 and 10 carbons, and peaking at 8. These are the products of pyrolysis of the cross-linked network, where C–C and C–O–C bonds have established among originally unsaturated molecules upon curing^14,44^. The second part of the pyrograms, which usually feature fatty acids naturally present in the oil (palmitic, oleic and stearic acids), were quite different. In particular the silylation of acidic moieties was only partially achieved in the WS sample, while it was fully successful in the NWS sample. Several replicate analyses were carried out, however the results remained unchanged.

### SSNMR

#### Low-resolution SSNMR experiments

**Figure 5:**
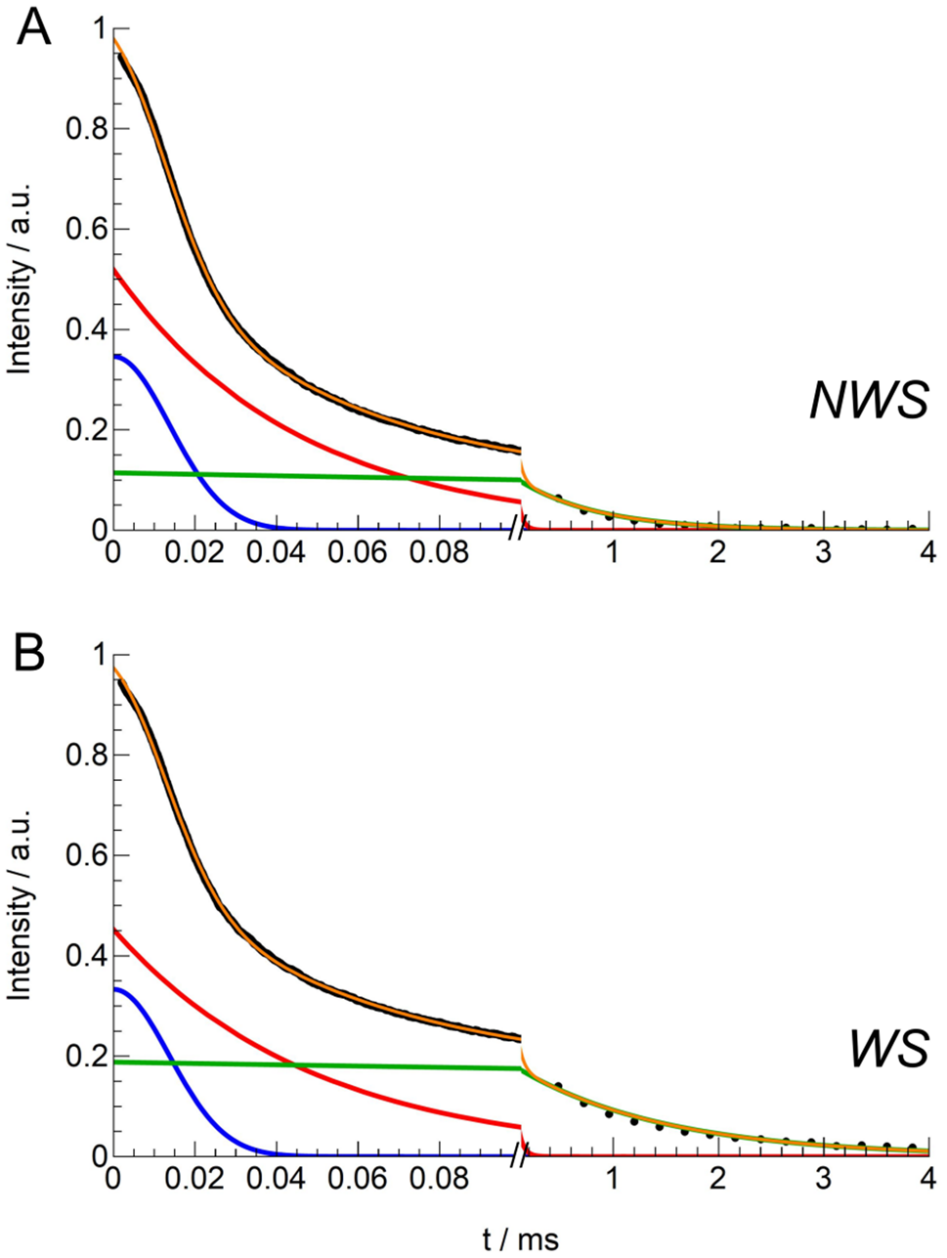
Fitting of the combined MSE/CPMG relaxation curves obtained for the (**a**) NWS and (**b**) WS sample. For each sample, the total fitting function (orange line) and the single contributions of the Gaussian (blue line), short-T_2_ (red line), and long-T_2_ (green line) exponential functions are shown.

^1^H Low-Resolution SSNMR (LR-SSNMR) experiments were carried out on WS and NWS to obtain information on the relative content of the mobile and cross-linked network as well as on their degree of mobility^48–50,77^. For each sample, ^1^H T_2_ relaxation times were measured by combining two experiments^77^: Magic Sandwich Echo (MSE)^78^, a convenient way to identify and quantify protons in rigid environments displaying a strong homonuclear dipolar coupling and a short T_2_, and Carr-Purcell-Meiboom-Gill (CPMG) experiments, useful to detect protons from mobile environments displaying longer T_2_’s. The discrete analysis of the combined MSE/CPMG relaxation curves (see SI- *Materials and Methods-SSNMR*) enabled the identification and quantification of domains with different mobility (classified as *rigid* and *mobile*), by fitting the relaxation curves with a linear combination of three different functions, each characterized by a T_2_ (T_2_^i^) and a weight percentage (W^i^%). Specifically, one Gaussian function (gau) and two exponential functions (exp1, exp2) were used to reproduce the decays, associated with protons in rigid (T_2_ ∼ 10–50 μs) or mobile (T_2_ > 100 μs) environments, respectively (Fig. 5).

The values of weight (W) and spin–spin relaxation time (T_2_) obtained by the fitting procedure are shown in Table 1. For each function, the values of ^1^H T_2_ and weight (W) are reported. The error on the values of T_2_ and W was estimated to be less than 5%.

**Table 1:** Best-fitting parameters obtained from the fitting of the combined MSE/CPMG relaxation curves with a linear combination of one Gaussian function (gau) and two exponential functions (exp1, exp2).

Sample	gau^a^		exp1^a^		exp2^a^	
	W (%)	T_2_ (μs)	W (%)	T_2_ (μs)	W (%)	T_2_ (μs)
NWS	35	19	53	44	12	757
WS	34	19	47	49	19	1411

^a^For each function, the values of ^1^H T_2_ and weight (W) are reported. The error on the values of T_2_ and W was estimated to be less than 5%.

In both samples, gau and exp1 present short T_2_ time constants (within 50 μs), typical of protons in rigid environments. These functions account for about the 81–88% of protons and can be mainly ascribed to the cross-linked fractions of the sample. Longer T_2_ values are obtained for exp2, which arises from protons belonging to a more mobile fraction of the sample, most likely associated with the non cross-linked, low molecular weight fraction. The relaxation time found for exp2 almost doubles by passing from NWS (~ 700 μs) to WS (~ 1400 μs); moreover, this component has a slightly greater weight in the case of the WS sample.

#### High-resolution SSNMR experiments

^1^H and ^13^C High-Resolution SSNMR (HR-SSNMR) experiments based on Magic Angle Spinning (MAS) and High Power Decoupling (HPD) techniques were acquired to achieve a more detailed chemical characterization of the mobile and polymeric phases of the oil paint samples.

Narrow peaks between 1 and 8 ppm were identified in the ^1^H MAS spectra, ascribable to protons with longer T_2_’s and belonging to organic compounds in the mobile fraction of the samples (Figure S6). These peaks were narrower for WS. Peak assignment of ^1^H HR-SSNMR spectra was accomplished by comparison with literature data^58,59,61^ and reported in Table S.5. No significant differences in the composition of the mobile phases were observed between the samples.

HR-SSNMR of ^13^C nuclei was also employed for the structural characterization of the paint layers. Specifically, ^13^C nuclei belonging to the mobile fraction of the sample were detected by ^13^C direct excitation with background suppression (DEPTH) spectra^79^, acquired using a short recycle delay (2 s). In this experiment, carbon nuclei belonging to the mobile fraction were selected thanks to their short T_1_ spin–lattice relaxation time^80^.

**Figure 6:**
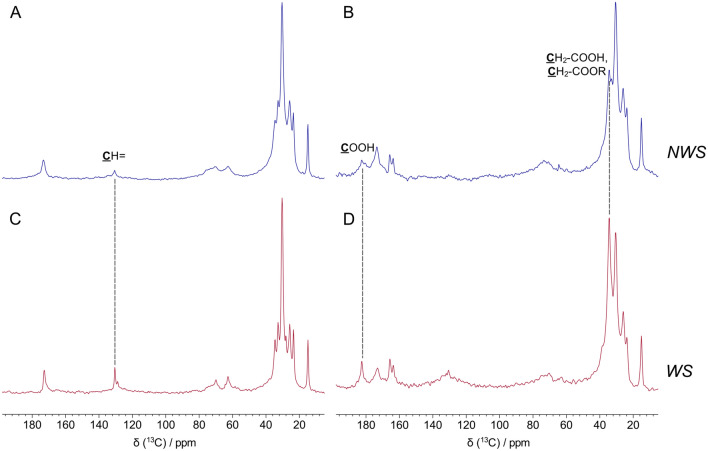
^13^C DEPTH (**a**,**c**) and ^1^H–^13^C CP-MAS (**b**,**d**) spectra of NWS (**a**,**b**) and WS (**c**,**d**) samples.

Similar spectra were obtained for the two samples (Fig. 6a,c; Figure S7a,c), both presenting the typical signals of unsaturated fatty acid glycerides. Detailed interpretation of the spectra, accomplished by comparison with literature data^58,59^, is reported in Table S.6. Narrower line shapes were noted for WS, in agreement with LR-SSNMR and ^1^H MAS results. Importantly, slightly higher signals were present in the WS spectrum at ~ 130 ppm, in the region of olefin carbons (peak integrals relative to the total spectrum integral: 3.5% for NWS; 6.8% for WS).

^1^H–^13^C CP-MAS spectra were also acquired. Since the cross-polarization process is efficient only for carbon-13 nuclei in closest proximity to protons in rigid environments, this experiment was useful to characterize the cross-linked fraction of the two samples. Concerning these experiments, we have made the assumption that the magnetization transfer efficiency is similar for the two samples; in fact, as shown by low resolution experiment, protons in rigid environments, which can be associated with the cross-linked fraction of the sample and which are the main responsible for the cross-polarization process, present very similar T_2_ values (NWS: 19 μs, 44 μs; WS: 19 μs, 49 μs); Therefore, based on this consideration, a qualitative comparison between the two ^1^H–^13^C CP-MAS spectra was made.

The spectral features of aliphatic chains can be observed from both spectra (Fig. 6b,d; Figure S7b,d), with the complete chemical assignment reported in Table S.6^58,59^. Contrary to the DEPTH spectra, in this case it is possible to observe quaternary carbon resonances from carbonyl groups (both acids and metal soaps) at 183 ppm, which may derive either from esters hydrolysis or from chain scission and oxidation reactions occurring within the polymeric network. This peak is slightly more intense for the WS sample. Furthermore, the WS spectrum presents a significantly higher signal in the aliphatic region at ~ 34 ppm, which can be assigned to methylene carbons in alpha position to C(=O)O– groups (**C**H_2_–COOH, **C**H_2_–COOR, **C**H_2_–COOM). The concomitant higher intensity of the two signals at 183 ppm and at 34 ppm observed for WS should not be ascribed to ester hydrolysis occurring to a greater extent in WS than in NWS; indeed, if this were the case, only the signal at 183 ppm should be higher in intensity, but not that at 34 ppm. Therefore, the increase of intensity of both signals in the WS spectrum may be ascribed to the formation of *new* C(=O)O– groups from oxidative chain scission reactions during the ageing process, which occurs to a lesser extent in the NWS sample.

## Discussion

The W&N ultramarine paint used in this study consists of synthetic ultramarine blue pigment mixed with linseed oil as main binder, with smaller amounts of safflower oil (HPLC-MS). The paint contains metal soaps (heptadecanoic acid was detected by GC-MS), as well as small amounts of hydroxymagnesite extender and kaolin (FTIR, SEM-EDX). The presence of hydrated aluminium oxide could not be excluded based on the FTIR analysis alone. The presence of free fatty acids in the paint formulation cannot be proven, although highly possible given their presence in the 1965 formulation (Table S.1) and the known widespread use of free fatty acids as wetting agents in modern oil paint formulations^81^.

Two model paints were prepared using the W&N ultramarine tube paint. After an initial drying period of 45 days, one of the model paint films was kept under ambient conditions and remained non-water sensitive (NWS model paint), and another was artificially aged in the presence of high lux and high relative humidity, which resulted in the paint being water sensitive (WS model paint). The paints were then allowed to naturally age for 2 years.

TG analysis showed that both NWS and WS samples contained very small amounts of moisture, at below 2%.

Under exposure to high relative humidity, the hydration of the hydromagnesite extender changed (FTIR), however evidence for the formation of epsomite was not found using infrared spectroscopy. It has been demonstrated that high relative humidity can promote the formation of epsomite (magnesium sulphate heptahydrate) and sodium sulphate in W&N French Ultramarine AOC oil paint^72^. Epsomite is a known cause of water sensitivity^69^ as the crystals are extremely water soluble. However if small amounts of epsomite were formed on the paint surface after artificial ageing, these were below the ATR detection limit, making it quite unlikely that these soluble sulphates were the cause of the water sensitivity observed: a fairly significant swelling effect during cleaning tests was observed, which is suggestive of rapid water ingress to the paint.

Artificial ageing under high relative humidity caused formation of amorphous metal soaps^18,19^—possibly, Mg salts—given concomitant changes to the hydromagnesite IR absorptions. Furthermore NWS presented a relatively higher content of unreacted free acids (HPLC). Soaps of alkaline earth metals are highly insoluble^37^, and metal soaps in general are believed to contribute to the stability of the paint film by forming an ionomer-like network^21,22^. The water sensitivity cannot thus be ascribed to the formation of metal soaps.

MS analyses (and ^13^C DEPTH NMR) revealed that WS contains higher amounts of unreacted double bonds (GC-MS), and both paints still contain di- and triglycerides (HPLC-MS). Moreover, pyrolysis data clearly indicated differences among the samples which influenced their reactivity towards the silylating agent (HMDS). Since the pigment is the same in the WS and NWS paints, and since the water content is also similarly very low in both samples (TGA), the differing analytical behaviour towards silylation must be ascribed to the molecular structure of the cross-linked network, as the soluble and hydrolysable fractions appear quite similar. A high concentration of alcoholic and acidic moieties might affect the derivatisation yield during in-situ pyrolysis and silylation, suggesting that the WS sample might feature a higher concentration of polar functional groups.

SSNMR showed that the paint layers presented two main fractions which were characterised by a different degree of mobility: a higher mobility phase and a rigid phase. The mobile phases contain both monomeric compounds (free fatty acids, mono-, di- and triglycerides), together with relatively small oligomers deriving from the initial stages of the cross-linking reactions. The rigid phase is the cross-linked network. The relative amounts, mobility level and chemical structure of these phases were different in the two model paint layers.

Based on the number of protons, a rough estimation of the relative content of mobile and crosslinked fractions of the two paint layers can be made. In WS, the mobile fraction comprises about 20% of the organic binder, while the cross-linked fraction is about 80%. In NWS—the control sample, the mobile fraction constitutes about 12% of the binder, and the cross-linked fraction is about 88%.

The mobile fraction presents a higher degree of mobility in the WS paint layer (LR-SSNMR, HR-SSNMR ^1^H MAS). The difference in mobility of the mobile phases may be interpreted in terms of their chemical composition: the mobile fraction of the WS sample contains more low-molecular weight compounds, i.e. more monomers, while the NWS one has more oligomers. Moreover, the mobile phase of the WS paint layer contains more unreacted double bonds (HR-SSNMR of ^13^C data).

Finally ^1^H–^13^C CP-MAS showed that the cross-linked fraction of the water sensitive sample has more C(=O)O– moieties (as free acidic moieties and in the form of metal carboxylates) covalently bound to the polymeric network, which should not arise from the hydrolysis of glycerides. This suggests that the higher amount of carboxylic functional groups covalently bound to the polymeric network found in the water sensitive paint layer might be ascribed to newly formed C(=O)O– groups deriving from the oxidative degradation of unsaturated glycerides.

## Conclusions

It has been hypothesized that when oxidative degradation of the paint film overwhelms cross-linking reactions, conservation issues, such as water sensitivity, may quickly establish. In this study high relative humidity—which was shown to favour oxidation reactions—was used to artificially age a model paint layer, which became water sensitive as a result. The paints were based on linseed and minor amounts of safflower oils mixed with ultramarine blue, a pigment which has been shown to promote high levels of oil oxidation, especially in combination with safflower oil. The analyses, with the fundamental contribution of LR-SSNMR experiments together with ^1^H MAS, ^13^C DEPTH and ^1^H–^13^C CP-MAS HR-SSNMR, revealed that the artificially aged sample was in a less advanced stage of curing, and the main chemical differences among the water sensitive model paint layer and the control non-water sensitive sample pertained to the cross-linked fractions. In particular, the water sensitive sample has a relatively higher content of monomers and oligomers, and a lower content of a cross-linked network—which is highly oxidised. When a paint in which the pigment particles are bound together by a relatively small cross-linked fraction containing several polar moieties, is subjected to surface cleaning using water, it is to be expected that exposure to water may swell the paint. In these conditions, the cohesive forces that keep the pigments anchored in the paint layer are disrupted by the interactions established between the polar oil network and the water. This would account for the typical phenomenology observable during attempts to surface-cleaning water sensitive paints: pigment loss, gloss change and surface disruption.

The study proves that the chemical speciation of the oil binder, and the chemical characterisation of all its fractions is of fundamental importance to understand the behaviour of oil paint films. In this context, the use of solid state NMR has proven crucial in revealing important details on the molecular mobility and the chemical structure of the mobile and cross-linked fractions of the paint binder, which are not available for molecular analysis to any of the conventional techniques classically used in the field. The methodology implemented in this work can be used to explore other pigmented oil paints and brands to help establish a wider understanding of oil paints and their conservation issues.

## Materials and methods

### Model paints

Two model paint films were prepared using W&N French Ultramarine Artists’ Oil Colour tube paint purchased in 2017. A custom-made film caster (Sheen Instruments) was used to create uniform paint films with a wet-film paint thickness of 400 μm. Commercially prepared oil primed canvas was used as the support [Belle Arti: 536 Medium Fine Linen, 508gm, from Jackson’s Art Supplies, UK]. After an initial 45 days of initial drying, one paint film continued to be maintained at ambient laboratory conditions, whilst the second model paint film was artificially aged for 52 days, using a Sanyo MCR-351H environmental chamber fitted with Philips TL-D/840 super 80, 58-W, long tubes, 4000 K bulbs with the ultraviolet (UV) component filtered out. Conditions inside the chamber were: 30 °C, 75% RH, and ~ 3500 lx at the paint surface. Both paint films were then left for a further two years of natural ageing under ambient laboratory conditions prior to this investigation.

### Water sensitivity tests

Water sensitivity tests were carried out using machine-prepared cotton swabs dampened with deionised water. As in previous studies^46,66^ water sensitivity was evaluated by rolling the swab over the paint surface until either a maximum of 50 swab rolls was reached without any noticeable surface-change, or until pigment/binder loss was observed.

### TGA

A TA Instruments Thermobalance model Q5000IR was used. Measurements were performed at a rate of 10 °C/min, from 25 to 900 °C under nitrogen flow (25 ml/min). The amount of sample in each TG measurement was about 4 mg. Temperature calibration was based on the Curie point of paramagnetic metals. A multipoint calibration with five Curie points from reference materials (Alumel, Ni, Ni83%Co17%, Ni63%Co37%, Ni37%Co63%) was performed.

### EDX

EDX was carried out using a LEO 1455VP SEM, operating conditions were: 20 kV, 15 mm working distance, 40Ps air. Samples were not coated.

### ATR-FTIR

A Thermo scientific Nicolet iZ10 benchtop ATR fitted with a germanium crystal of was used for surface analysis of the sample. Data was acquired with 64 scans and a resolution of 4 cm^−1^ across a wavenumber range of 4000 to 600 cm^−1^. Spectra were processed using Omnic 9 software. Three ATR spectra were acquired per sample. Average of three spectra were used for comparisons between artificially aged water sensitive samples and control non water sensitive samples.

### FTIR bulk

A Thermo scientific Nicolet iN10 MX microscope with a single diamond cell, equipped with an MCT-A/CdTe detector. Data was acquired with 64 scans and a resolution of 4. Spectra were processed using Omnic 8. Three spectra were acquired per sample. Average of three spectra were used for comparisons between artificially aged samples and control samples.

### GC-MS

A gas chromatograph 6890 N GC System (Agilent Technologies, Palo Alto, CA, USA) was used, coupled with a 5975 Mass Selective Detector (Agilent Technologies, Palo Alto, CA, USA) single quadrupole mass spectrometer. The chromatograph was equipped with a HP-5 ms fused silica capillary column (5%-diphenyl-95%-dimethyl polysiloxane, 30 m, 0.25 mm i.d., 0.25 mm film thickness (J&W Scientific, Agilent Technologies, Palo Alto, CA) connected with a deactivated silica pre-column (2 m, 0.32 mm i.d., (J&WScientific Agilent Technologies, Palo Alto, CA). The PTV injector, used in splitless mode, was kept at 280 °C. The carrier gas was used in the constant flow mode (He, purity 99.995%) at 1.0 mL/min. The chromatographic oven was programmed as follows: initial temperature 80 °C, isothermal for 2 min; 10 °C-min up to 280 °C, and isothermal for 30 min. The MS transfer line temperature was 280 °C; the MS ion source temperature was 230 °C, and the MS quadrupole temperature was 150 °C. The mass spectrometer operated in EI positive mode (70 eV) with a scan range *m-z* 50e700. MS spectra were recorded both in TIC (total ion current) and SIM (single ion monitoring) mode. Samples were analysed in triplicate, after saponification, hydrolysis and silylation. Quantitations were based on calibration curves. Details of the analytical procedure and data analysis are reported elsewhere^75^.

### Py-GC-MS

The pyrolyser used was a micro-furnace Multi-Shot Pyrolyzer EGA-Py-3030D (Frontier Lab) coupled to a gas chromatograph 6890 (Agilent Technologies, Palo Alto, CA, USA) and to an Agilent 5973 Mass Selective Detector operating in electron impact mode (EI) at 70 eV. The split-splitless injector was used in split mode at 280 °C, with a split ratio 20:1. The chromatographic conditions were as follows: 50 °C isothermal for 2 min, 10 °C-min up to 280 °C and isothermal for 2 min, 15 °C-min up to 300 °C and isothermal for 30 min. The carrier gas (He, purity 99.9995%) was used in the constant flow mode at 1.0 ml/min. The temperatures of the MS transfer line was 280 °C, MS ion source was 230 °C and MS quadrupole was 150 °C. The mass spectrometer was operated in EI positive mode (70 eV) with a scan range m-z 50–600. MS spectra were recorded in TIC (total ion current mode). Samples (200–300 μg) were placed in a pyrolysis cup and admixed to 5 µL 1,1,1,3,3,3-hexamethyldisilazane (HMDS) used as a silylating agent for the in-situ derivatisation of pyrolysis products. Analyses were performed in triplicate.

### HPLC-ESI-Q-TOF

HPLC-ESI-Q-ToF analyses were carried out using a 1200 Infinity HPLC, coupled with a Quadrupole-Time of Flight tandem mass spectrometer 6530 Infinity Q-ToF detector by a Jet Stream ESI interface (Agilent Technologies). The chromatographic separation was carried out using a Poroshell 120 EC-C18 column with a Zorbax eclipse plus C-18 guard column at a flow rate of 0.3 mL·min^−1^ and at 45 °C. Aliquots of 10 µL were injected and the elution gradient was programmed using methanol–water 85:15 (eluent A) and *iso*-propanol (eluent B) as follows: 90% A for 5 min, followed by a linear gradient to 90% B in 30 min (held for 10 min). Re-equilibration time for each analysis run was 10 min.

ESI operating conditions: drying gas (N_2_, purity > 98%): 350 °C and 10 L·min^−1^; capillary voltage 4.5 kV; nebulizer gas 35 psig; sheath gas (N_2_, purity > 98%): 375 °C and 11 L·min^−1^. High resolution MS and MS–MS spectra were acquired in negative mode for the first 10 min of the chromatographic run, while was switch to positive mode until the end of the run. The acquisition was performed in the range 100–1700 m-z The fragmentor was kept at 200 V, nozzle voltage 1000 V, skimmer 65 V, octapole RF 750 V. The collision gas was nitrogen (purity 99.999%).

Samples (0.1 mg) were subjected to extraction with a chloroform-hexane mixture, dried and redissolved in the elution mixture, and filtered PTFE filter. Samples were analysed in triplicate. More details are reported elsewhere^82^.

### Solid state NMR (SSNMR)

^1^H Time Domain Low-Resolution Solid-State NMR (LR-SSNMR) measurements were performed under low-resolution conditions working at a Larmor frequency of 20.8 MHz using a Niumag permanent magnet interfaced with a Stelar PC-NMR console. A 5 mm probe was used with a ^1^H 90° pulse duration of τ_90_ = 3 μs for all samples. On-resonance Free Induction Decays (FIDs) were recorded for all samples using the Magic Sandwich Echo (MSE) pulse sequence^78^ with a total echo duration of τ_MSE_ = 6 (4τ_φ_ + 2τ_90_) = 72 μs, with τ_φ_ = 1.5 μs, using a dwell time of 0.1 μs and 8 k acquisition points; 1000 scans were accumulated using a recycle delay (RD) of 0.5 s, sufficient to guarantee quantitative measurements. Alternating-phase Carr-Purcell-Meiboom-Gill (CPMG) experiments (pulse sequence: RD − (π/2)_x_ − [τ − (π)_y_ − 2τ − (π)_-y_ − 2τ − (π)_-y_ − 2τ − (π)_y_ − τ]_n_) were also acquired on both samples; 1000 transients were accumulated using a recycle delay of 0.5 s, an echo delay τ of 27 μs and acquiring 300 data points. The first 1000 data points of the MSE ^1^H FID (recorded within the first 100 μs of the acquisition) were joined together with the data points of the CPMG experiment. The resulting combined MSE/CPMG relaxation curves were analyzed by a discrete approach using a non-linear least square fitting procedure implemented in the Mathematica® environment (Wolfram Research Europe Ltd, Oxfordshire, United Kingdom).

High-Resolution ^1^H and ^13^C Solid-State NMR (HR-SSNMR) spectra of the oil paint samples were acquired on a Varian InfinityPlus spectrometer working at a Larmor frequency of 400.34 and 100.67 MHz for ^1^H and ^13^C, respectively, using a 3.2 mm Cross Polarization (CP) – Magic Angle Spinning (MAS) probe. All experiments were recorded using a MAS frequency of 15 kHz. ^1^H MAS spectra were recorded acquiring 8000 points and using a recycle delay of 2 s, a dwell time of 5 μs and 32 transients for spectra accumulation.

^1^H–^13^C Cross Polarization-Magic Angle Spinning (CP-MAS) spectra were recorded using a contact time of 3 ms with a linear ramp; 27,000–31,000 transients were accumulated, using a recycle delay of 2 s between consecutive transients and a dwell time of 20 μs. A reference spectrum of a silica filled rotor was acquired exactly in the same conditions and subtracted from the ^1^H–^13^C CP-MAS experiments, to eliminate signals ascribable to the background. ^13^C direct excitation with background suppression (DEPTH)^79^ experiments were recorded accumulating 12,000–30,000 transients, using a recycle delay of 2 s and a dwell time of 20 μs. Both ^1^H–^13^C CP-MAS and ^13^C DEPTH spectra were acquired under High-Power Decoupling (HPD) from ^1^H nuclei. ^13^C chemical shifts were referred to hexamethylbenzene and TMS as secondary and primary references, respectively.

Data processing and analysis of all HR-SSNMR spectra were performed using MestReNova 12.0.4 (Mestrelab Research).

## Supplementary Information

Supplementary Information.

## Data Availability

The data generated and analysed during this study which are not already included in this published article (and its Supplementary Information file) are available from the corresponding author on reasonable request.

## References

[1] Bonaduce I (2019). Conservation issues of modern oil paintings: a molecular model on paint curing. Acc. Chem. Res..

[2] Tumosa CS, Mecklenburg MF (2005). The influence of lead ions on the drying of oils. Stud. Conserv..

[3] Soucek M, Khattab T, Wu J (2012). Review of autoxidation and driers. Prog. Org. Coat..

[4] Frankel EN (2012). Lipid Oxidation.

[5] Honzíček J (2019). Curing of air-drying paints: a critical review. Ind. Eng. Chem. Res..

[6] Shahidi F (2005). Bailey's Industrial Oil and Fat Products, Edible Oil and Fat Products: Processing Technologies.

[7] Oakley LH, Casadio F, Shull PKR, Broadbelt PLJ (2018). Modeling the evolution of crosslinked and extractable material in an oil-based paint model system. Angew. Chem. Int. Ed..

[8] Frankel EN (1982). Volatile lipid oxidation products. Prog. Lipid Res..

[9] Boon, J., Peulvé, S., van den Brink, O., Duursma, M. & Rainford, D. in Early Italian Paintings : Techniques and Analysis, Symposium. (eds T. Bakkenist, R. Hoppenbrouwers, & H. Dubois) (Limburg Conservation Institute).

[10] Van Den Berg, J. D., Van Den Berg, K. J. & Boon, J. J. in ICOM-CC 12th Triennial meeting. 248-253 (James and James).

[11] Erhardt D, Tumosa CS, Mecklenburg MF (2005). Long-term chemical and physical processes in oil paint films. Stud. Conserv..

[12] Hermans JJ, Keune K, Loon Av, Iedema PD (2016). The crystallization of metal soaps and fatty acids in oil paint model systems. Phys. Chem. Chem. Phys..

[13] Baij L, Chassouant L, Hermans JJ, Keune K, Iedema PD (2019). The concentration and origins of carboxylic acid groups in oil paint. RSC Adv..

[14] La Nasa J (2019). The role of the polymeric network in the water sensitivity of modern oil paints. Sci. Rep..

[15] Lazzari M, Chiantore O (1999). Drying and oxidative degradation of linseed oil. Polym. Degrad. Stab..

[16] Tumosa CS, Mecklenburg MF (2003). Weight changes on oxidation of drying and semi-drying oils. Collection Forum.

[17] Ma X (2019). Revealing the distribution of metal carboxylates in oil paint from the micro- to nanoscale. Angew. Chem. Int. Ed..

[18] Garrappa, S., Kočí, E., Švarcová, S., Bezdička, P. & Hradil, D. Initial stages of metal soapsformation in model paints: the role of humidity. *Microchem. J.* **156**, 104842 (2020).

[19] Possenti E, Colombo C, Realini M, Song CL, Kazarian SG (2020). Insight into the effects of moisture and layer build-up on the formation of lead soaps using micro-ATR-FTIR spectroscopic imaging of complex painted stratigraphies. Anal. Bioanal. Chem..

[20] Mazzeo R (2008). Attenuated total reflection micro FTIR characterisation of pigment–binder interaction in reconstructed paint films. Anal. Bioanal. Chem..

[21] Hermans JJ, Keune K, van Loon A, Corkery RW, Iedema PD (2016). Ionomer-like structure in mature oil paint binding media. RSC Adv..

[22] Boon, J. J., Hoogland, F., Keune, K. & Parkin, H. M. in AIC Paintings Specialty Group Postprints. (ed Helen Mar Parkin) 16-23 (American Institute for Conservation of Historic & Artistic Works ).

[23] Beerse M, Keune K, Iedema P, Woutersen S, Hermans J (2020). Evolution of zinc carboxylate species in oil paint ionomers. ACS Appl. Polym. Mater..

[24] Meilunas RJ, Bentsen JG, Steinberg A (1990). Analysis of aged paint binders by FTIR spectroscopy. Stud. Conserv..

[25] Vagnini M (2009). FT-NIR spectroscopy for non-invasive identification of natural polymers and resins in easel paintings. Anal. Bioanal. Chem..

[26] Van der Weerd J, Van Loon A, Boon JJ (2005). FTIR studies of the effects of pigments on the aging of oil. Stud. Conserv..

[27] Piqué F, Chiari G, Colombini MP, Torraca G, Biscontin G, Driussi G (2010). Scienza e Beni Culturali.

[28] Bonaduce I, Andreotti A, Colombini MP, Modugno F (2009). Organic mass spectrometry in art and archaeology.

[29] La Nasa J, Modugno F, Degano I (2020). Liquid chromatography and mass spectrometry for the analysis of acylglycerols in art and archeology. Mass Spectrom. Rev..

[30] Zumbühl S, Scherrer NC, Eggenberger U (2014). Derivatization technique to increase the spectral selectivity of two-dimensional Fourier transform infrared focal plane array imaging: analysis of binder composition in aged oil and tempera paint. Appl. Spectrosc..

[31] Berg JDJvd, Berg KJvd, Boon JJ (2001). Determination of the degree of hydrolysis of oil paint samples using a two-step derivatisation method and on-column GC/MS. Prog. Org. Coat..

[32] La Nasa J, Modugno F, Aloisi M, Lluveras-Tenorio A, Bonaduce I (2018). Development of a GC/MS method for the qualitative and quantitative analysis of mixtures of free fatty acids and metal soaps in paint samples. Anal. Chim. Acta.

[33] Modugno F (2019). On the influence of relative humidity on the oxidation and hydrolysis of fresh and aged oil paints. Sci. Rep..

[34] Artesani A (2020). Zinc oxide instability in drying oil paint. Mater. Chem. Phys..

[35] Catalano J (2020). Review of the use of NMR spectroscopy to investigate structure, reactivity, and dynamics of lead soap formation in paintings. Magn. Reson. Chem..

[36] Otero V (2014). Characterisation of metal carboxylates by Raman and infrared spectroscopy in works of art. J. Raman Spectrosc..

[37] Robinet L, Corbeil MC (2003). The characterization of metal soaps. Stud. Conserv..

[38] Hermans JJ, Keune K, van Loon A, Iedema PD (2015). An infrared spectroscopic study of the nature of zinc carboxylates in oil paintings. J. Anal. At. Spectrom..

[39] Cotte M, Checroun E, Susini J, Walter P (2007). Micro-analytical study of interactions between oil and lead compounds in paintings. Appl. Phys. A Solids Surf..

[40] MacDonald MG, Palmer MR, Suchomel MR, Berrie BH (2016). Reaction of Pb (II) and Zn (II) with ethyl linoleate to form structured hybrid inorganic–organic complexes: a model for degradation in historic paint films. ACS Omega.

[41] Keune K, Boon JJ (2007). Analytical imaging studies of cross-sections of paintings affected by lead soap aggregate formation. Stud. Conserv..

[42] Muizebelt W, Hubert J, Venderbosch R (1994). Mechanistic study of drying of alkyd resins using ethyl linoleate as a model substance. Prog. Org. Coat..

[43] Degano I, Modugno F, Bonaduce I, Ribechini E, Colombini MP (2018). Recent advances in analytical pyrolysis to investigate organic materials in heritage science. Angew. Chem. Int. Ed..

[44] Pizzimenti, S. *et al.* Oxidation and cross-linking in the curing of air-drying artists' oil paints. *ACS Appl. Polym. Mater.* (2021) **3**, 1912–1922 (2021).

[45] Vereshchagin A, Novitskaya GV (1965). The triglyceride composition of linseed oil. J. Am. Oil Chem. Soc..

[46] Lee J (2018). Scientific investigation into the water sensitivity of twentieth century oil paints. Microchem. J..

[47] Bronken IAT, Boon JJ, Burnstock A (2014). Issues in Contemporary Oil Paint.

[48] Hughes DJ (2018). Phase separation in amorphous hydrophobically modified starch–sucrose blends: glass transition, matrix dynamics and phase behavior. Carbohydr. Polym..

[49] Martini F, Borsacchi S, Geppi M, Ruggeri G, Pucci A (2014). Understanding the aggregation of bis (benzoxazolyl) stilbene in PLA/PBS blends: a combined spectrofluorimetric, calorimetric and solid state NMR approach. Polym. Chem..

[50] Borsacchi S (2018). Rubber-filler interactions in polyisoprene filled with in situ generated silica: a solid state NMR study. Polymers.

[51] Carignani E (2020). Effect of sepiolite treatments on the oxidation of sepiolite/natural rubber nanocomposites prepared by latex compounding technique. Appl. Clay Sci..

[52] Proietti N, Capitani D, Di Tullio V (2018). Nuclear magnetic resonance, a powerful tool in cultural heritage. Magnetochemistry.

[53] Spyros A, Webb GA (2016). Modern Magnetic Resonance.

[54] Rehorn C, Blümich B (2018). Cultural heritage studies with mobile NMR. Angew. Chem. Int. Ed..

[55] Udell NA, Hodgkins RE, Berrie BH, Meldrum T (2017). Physical and chemical properties of traditional and water-mixable oil paints assessed using single-sided NMR. Microchem. J..

[56] Di Tullio V (2020). Water diffusion and transport in oil paints as studied by unilateral NMR and 1H high-resolution MAS-NMR spectroscopy. ChemPhysChem.

[57] Catalano J (2018). Molecular dynamics of palmitic acid and lead palmitate in cross-linked linseed oil films: implications from deuterium magnetic resonance for lead soap formation in traditional oil paintings. Solid. State Nucl. Mag..

[58] Kehlet C, Kuvvetli F, Catalano A, Dittmer J (2016). Solid-state NMR for the study of Asger Jorn’s paintings. Microchem. J..

[59] Spyros A, Anglos D (2004). Study of aging in oil paintings by 1D and 2D NMR spectroscopy. Anal. Chem..

[60] Spyros A, Anglos D (2006). Studies of organic paint binders by NMR spectroscopy. Appl. Phys. A Solids Surf..

[61] Cipriani G (2009). Recent advances in swollen-state NMR spectroscopy for the study of drying oils. J. Cult. Heritage.

[62] de la Rie ER, Michelin A, Ngako M, Del Federico E, Del Grosso C (2017). Photo-catalytic degradation of binding media of ultramarine blue containing paint layers: A new perspective on the phenomenon of “ultramarine disease” in paintings. Polym. Degrad. Stab..

[63] Schnetz K (2020). Evidence for the catalytic properties of ultramarine pigment. J. Cult. Herit..

[64] Cato E, Borca C, Huthwelker T, Ferreira ES (2016). Aluminium X-ray absorption near-edge spectroscopy analysis of discoloured ultramarine blue in 20th century oil paintings. Microchem. J..

[65] Ormsby B, Lee J, Bonaduce I, Lluveras-Tenorio A, van den Berg KJ (2019). Conservation of modern oil paintings.

[66] Lee J, Ormsby B, Burnstock A, van den Berg KJ, van den Berg KJ (2019). Conservation of Modern Oil Paintings.

[67] Baij L, Hermans JJ, Keune K, Iedema PD (2018). Time-dependent ATR-FTIR spectroscopic studies on solvent diffusion and film swelling in oil paint model systems. Macromolecules.

[68] Helwig K, Moffatt EA, Corbeil M-C, Duguay D (2015). Early twentieth-century artists’ paints in toronto: archival and material evidence. J. Can. Assoc. Conserv. (CAC).

[69] Silvester G (2014). A cause of water-sensitivity in modern oil paint films: The formation of magnesium sulphate. Stud. Conserv..

[70] Kuenzel C, Zhang F, Ferrandiz-Mas V, Cheeseman C, Gartner E (2018). The mechanism of hydration of MgO-hydromagnesite blends. Cem. Concr. Res..

[71] Smith BC (2018). Infrared spectral interpretation: a systematic approach.

[72] Harrison, J. *Investigation of the influence of light and relative humidity on the formation of magnesium sulphate heptahydrate in Cadmium Yellow and French Ultramarine oil paint films* Masters thesis, Imperial College London, Department of Materials, (2020).

[73] Hollingbery L, Hull T (2010). The thermal decomposition of huntite and hydromagnesite—a review. Thermochim. Acta.

[74] Strekopytov S, Exley C (2006). Thermal analyses of aluminium hydroxide and hydroxyaluminosilicates. Polyhedron.

[75] Bonaduce I (2012). New insights into the ageing of linseed oil paint binder: a qualitative and quantitative analytical study. PLoS ONE.

[76] Chiavari G, Fabbri D, Prati S (2005). Effect of pigments on the analysis of fatty acids in siccative oils by pyrolysis methylation and silylation. J. Anal. Appl. Pyrolysis.

[77] Martini F (2019). Molecular dynamics of amphiphilic random copolymers in the bulk: a 1H and 19F NMR relaxometry study. Macromol. Chem. Phys..

[78] Maus A, Hertlein C, Saalwächter K (2006). A robust proton NMR method to investigate hard/soft ratios, crystallinity, and component mobility in polymers. Macromol. Chem. Phys..

[79] Cory D, Ritchey W (1988). Suppression of signals from the probe in Bloch decay spectra. J. Magn. Reson..

[80] Martini F (2020). Structural order and NIR reflective properties of perylene bisimide pigments: Experimental evidences from a combined multi-technique study. Dyes Pigm..

[81] Izzo, F. C., van den Berg, K. J., van Keulen, H., Ferriani, B. & Zendri, E. in *Issues in Contemporary Oil Paint* (eds A. Burnstock *et al.*) 75–104 (Springer, 2014).

[82] Banti D (2018). A molecular study of modern oil paintings: investigating the role of dicarboxylic acids in the water sensitivity of modern oil paints. RSC Adv..

