# Supplementary material for: The stability of paintings and the molecular structure of the oil paint polymeric network
Source: Sci Rep. 2021 Jul 9;11:14202. doi: 10.1038/s41598-021-93268-8 (PMC8270892; doi:10.1038/s41598-021-93268-8)
Supplement: Supplementary file 1 — Supplementary Information. [file 41598_2021_93268_MOESM1_ESM.docx]

**Supplementary information**

The stability of paintings and the molecular structure of the oil paint polymeric network

Francesca Nardelli^[a]^, Francesca Martini^[a,b]^, Judith Lee^[c]^, Anna Lluvears-Tenorio^[a]^, Jacopo La Nasa^[a]^, Celia Duce^[a]^, Bronwyn Ormsby^[c]^, Marco Geppi^[a,b]^, and Ilaria Bonaduce*^[a]^.

[a] Department of Chemistry and Industrial Chemistry, University of Pisa, Via Giuseppe Moruzzi 13, 56124, Pisa (IT).

[b] Centro per l’Integrazione della Strumentazione Scientifica dell’Università di Pisa (CISUP), Lungarno Pacinotti 43, 56126, Pisa (IT).

[c] Conservation Department, Tate, Millbank, London SW1P 4RG (UK)

**Composition of Winsor & Newton French Ultramarine Oil Paint: Historical 1965 formulation**

Composition of Winsor & Newton French Ultramarine Oil Paint: Historical 1965 formulation, obtained from The Winsor & Newton Archive, of the Hamilton Kerr Institute, Cambridge University, UK.

Table S. 1. 1965 W&N, Artists Oil Colour tube paint formulation for French Ultramarine. The synthetic ultramarine pigment used in the formulation was bought by W&N from external suppliers.

| *Component* | *Percentage (w/w)* |
| --- | --- |
| *Pigment* | *55.86%* |
| *Magnesium Carbonate* | *6.84%* |
| *Alumina White (hydrated aluminium oxide)* | *6.84%* |
| *Safflower Oil* | *23.83%* |
| *Fatty Acid* | *0.87%* |
| *Manganese (Mn) Drier* | *4.489* |
| *Magnesium Stearate* | *1.26%* |

***FTIR and SEM-EDX***

Figure S. 1. FTIR spectra of bulk paint sample control NWs sample (top) and WS sample (bottom).

Table S. 2. Band assignments for IR spectra

| Absorption bands (cm^-1^) | Assignment |
| --- | --- |
| ~3362-3392 (-OH stretch); 2928, 2855 (CH stretch); 1740-1 (ester carbonyl absorption); 1467 (CH_3_-O); 1458 (CH_3_ asymmetric bend); 1379 (CH_3_ umbrella mode); 1321-2 (C-O); 1250 (C-O ester bond); 1161-2 (C-O ester bond) | Oil |
| 1008 (Si-O-Si asymmetric stretch); 692-4, 656-9, 584 and 541 (all Si-O vibrations) | Ultramarine pigment |
| 1483, 1429-21 (CO_3_^2 -^ / HCO_3_^-^; v3 asymmetric stretching vibration); 1467 (C-O stretch, carbonate absorption); 884 (C-O out-of-plane bend, carbonate absorption) | Hydromagnesite [Mg_5_(CO_3_)_4_(OH)_2_] |
| 3694 (Al---O-H stretch); 3619 (O-H stretch; 1630 the H-O-H bending of water); 1377-9 (Al-O); 915 (Al-OH) | Kaolin |
| 1096 (Si-O-Si asymmetric stretch); 802 (Si-O-Si symmetric stretch); | Silica |
| 1640 broad (OH) ; 1321-2 (C-O stretch) | Possible metal oxalate |
| 1566 shoulder (COO^-^ asymmetric stretch) | Metal carboxylate |
| 1590 broad (COO^-^ asymmetric stretch) | Possible amorphous metal soaps |
| Broad band ~3400 and ~1633 (OH stretch and bending of coordinated H_2_O); broad band ~950 (Al-(OH)-Al bend) | Possible alumina hydrate extender [could be largely obscured by the ultramarine pigment absorptions] |


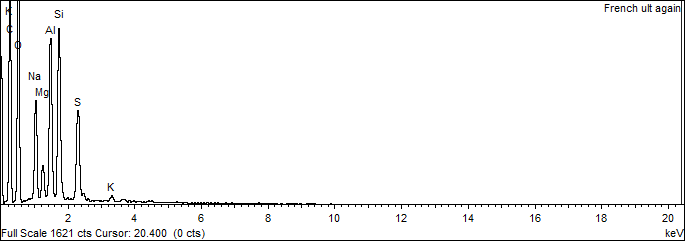


Figure S. 2. EDX spectra of W&N French Ultramarine paint. The profile of Na, Al, Si and S are characteristic of synthetic ultramarine pigment. Mg is associated with the hydromagnesite extender, and possibly magnesium carboxylates.

***TGA***





Figure S. 3: Thermogravimetric profile of ultramarine blue at 10°min^-1^ under nitrogen

***Mass spectrometry***

*GC-MS.* Figure S. 4 reports the chromatograms of samples NWS and WS, and Table S.3 reports the characteristic parameters used in the analysis of drying oils[^1^](#_ENREF_1), i.e the ratio between the relative content of palmitic and stearic acid (P/S), azelaic and palmitic acid (A/P), oleic and stearic acid (O/S), and the sum of the relative content of dicarboxylic acids (suberic acid, azelaic acid and sebacic acid) (∑ Dic.%).

Figure S. 4. GC-MS chromatograms of samples NWS and WS

Table S. 3. Characteristic parameters used in the analysis of drying oils by GC-MS: ratio between the relative content of palmitic and stearic acid (P/S), azelaic and palmitic acid (A/P), oleic and stearic acid (O/S), and the sum of the relative content of dicarboxylic acids (suberic acid, azelaic acid and sebacic acid) (∑ Dic.%).

| Sample | A/P | P/S | O/S | Σ Dic% |
| --- | --- | --- | --- | --- |
| NWS | 1.3 | 4.8 | 4.3 | 42% |
| WS | 1.1 | 4.7 | 5.4 | 36% |

*HPLC-MS*

Table S. 4. Acyl glycerols identified in the extracts of the paint samples after HPLC-ESI-MS analysis. A: arachidyl (C_20:0_), L: linoleyl (C_18:2_), O: oleyl (C_18:1_), S: stearyl (C_18:0_), P: palmityl (C_16:0_). For the oxidized acyl substituents: C_n_° of carbon atoms: n° of unsaturation, n° of OH.

| ***Class*** | ***Compound*** | ***m/z*** |
| --- | --- | --- |
| **OxDAGs** | OC_18:1(OH)_ | 659.5 |
|  | SC_18:1(OH)_ | 661.5 |
|  | SC_18(OH)_ | 663.5 |
|  | SC_18:1(2OH)_ | 677.5 |
|  | SC_18(2OH)_ | 679.5 |
| **OxTAGs** | POC_18:2(OH)_ | 895.7 |
|  | POC_18:1(OH)_ | 897.7 |
|  | PC_18:2(OH)_C_18:2(OH)_ | 909.7 |
|  | PC_18:1(OH)_C_18:2(OH)_ | 911.7 |
|  | PC_18:1(OH)_C_18:1(OH)_ | 913.7 |
|  | PC_18:1(OH)_C_18(OH)_ | 915.8 |
|  | PC_18 (OH)_C_18(OH)_ | 917.7 |
|  | OOC_18:2(OH)_ | 921.8 |
|  | OOC_18:1(OH)_ | 923.8 |
|  | OOC_18(OH)_ | 925.8 |
|  | C_18:2(OH)_C_18:2(OH)_S | 937.7 |
|  | C_18:2(OH)_C_18:1(OH)_S | 939.7 |
| **TAGs** | POP | 855.7 |
|  | OOP | 881.7 |
|  | OLS | 907.7 |
|  | PPS | 857.7 |
|  | OSP | 883.7 |
|  | OOO | 907.7 |
|  | PSS | 885.7 |
|  | OOS | 909.7 |
|  | OSS | 911.8 |
|  | SSS | 913.8 |
|  | ArSO | 939.8 |
|  | ArOO | 937.8 |

*Py-GC-MS*

Figure S. 5. Py-GC-MS chromatograms of samples NWS (top) and WS (bottom)

***SSNMR***

Figure S. 6 ^1^H MAS spectra of NWS (a) and WS (c). Spectral expansions of the resolved region for NWS (b) and WS (d).

Table S. 5. ^1^H MAS chemical shift assignment for WS and NWS samples. The chemical assignment refers to the nucleus highlighted in bold and underlined.

| **^1^H MAS** | |
| --- | --- |
| **Group assignment** | **δ (^1^H, ppm)** |
| C**H_3_** | 0.8 |
| (C**H_2_**)_n_ | 1.2 |
| C**H**-CH_2_,  C**H_2_**-CH= | 1.9 |
| C**H_2_**-COOH,  C**H_2_**-COO^-^,  C**H_2_**-COOR | 2.2 |
| C**H_2_**-O,  C**H**-O,  C**H_2_**-O-COR | 3.9-4.3 |
|  |  |
| C**H**=,  C**H**-O-COR | 5.3 |

Table S. 6 ^13^C chemical shift assignment for WS and NWS samples. The chemical assignment refers to the nucleus highlighted in bold and underlined.

| **^13^C DEPTH and ^1^H-^13^C CP-MAS** | |
| --- | --- |
| Group assignment | δ (^13^C, ppm) |
| **C**H_3_ | 14.9 |
| **C**H_2_-CH_3_ | 23.5 |
| =CH-**C**H_2_-CH= | 25.8 |
| **C**H_2_-CH= | 28.0 ^a^ |
| (**C**H_2_)_n_ | 30.4 |
| **C**H_2_-CH_2_-CH_3_ | 32.8 |
| **C**H_2_-COOH,  C**H_2_**-COO^-^,  **C**H_2_-COOR | 34.7 |
| **C**H_2_-O-COR,  **C**H_2_-O | 62.8 ^a^ |
| **C**H-O-COR,  **C**H-O | 70.0 |
| **C**H= | 128.8, 130.5 |
| *not assigned* | 163.6, 165.7 ^b^ |
| **C**OOR | 172.8 |
| **C**OOH,  **C**OO^-^ | 182.5 ^b^ |
|  |  |

^a^ Signals detected in the ^13^C DEPTH spectra only

^b^ Signals detected in the ^1^H-^13^C CP-MAS spectra only

Figure S. 7 Expansion of the aliphatic regions of the ^13^C DEPTH (a, c) and ^1^H-^13^C CP-MAS (b, d) spectra of NWS (a, b) and WS (c, d).

References

(1) Colombini, M. P.; Andreotti, A.; Bonaduce, I.; Modugno, F.; Ribechini, E.: Analytical Strategies for Characterizing Organic Paint Media Using Gas Chromatography/Mass Spectrometry. *Acc. Chem. Res.* 2010, *43*, 715-727.
